# Infectious Diseases Society for Obstetrics and Gynecology: Annual Scientific Meeting, August 9–11, 2001

**DOI:** 10.1155/S106474490100028X

**Published:** 2001


					Infectious Diseases Society for Obstetrics and Gynecology
Annual Scientific Meeting
August 9-11, 2001
Loews Le Concorde Hotel
Quebec City, Quebec, Canada
PROGRAM
Jointly sponsored by the American College
of Obstetricians and Gynecologists
(ACOG)
Infect Dis Obstet Gynecol 2001;9:155-190
155
I
N
F
E
C
T
I
O
U
S
D
I
S
E
A
S
E
S
S
O
CIETY FOR OBSTE
T
R
I
C
S
A
N
D
G
Y
N
E
C
O
L
O
G
Y
1973
Thursday, August 9, 2001
7:00 am - 3:00 pm Registration
Morning session
8:00 am Welcome - Sebastian Faro, MD, PhD, IDSOG President
Opening Remarks - David Soper, MD, Scientific Program Chair
8:10 am - 12:00 noon Symposium: Emerging Resistance in Infectious Diseases
8:10 - 9:10 am Presidential Guest Lecturer
Antifungal resistance in Candida
Jack D. Sobel, MD, Wayne State University, School of Medicine
9:10 - 9:15 am Q & A
9:15 - 9:30 am Break
9:30 - 10:15 am Resistance in bacteria
Barbara Murray, MD
Division of Infectious Diseases, University of Texas Medical School
at Houston
10:15 - 11:00 am Resistance in viruses
Douglas L. Mayers, MD, Division of Infectious Diseases,
Henry Ford Detroit Center
11:00 - 11:45 am Resistance in parasites including Chlamydia
George Schmid, MD, Centers for Disease Control, Atlanta, GA
11:45 - 12:00 noon Q & A
12:00 noon - 1:30 pm Break for Lunch (Lunch Will Be On Your Own)
Program
156 INFECTIOUS DISEASES IN OBSTETRICS AND GYNECOLOGY
Afternoon session
1:30 - 3:00 pm Scientific Session I Moderator: Jeff Peipert, MD
1:30 pm Symptoms associated with chlamydial infection among minority women
Tony S. Wen, MD
1:45 pm Lower genital tract infection and endometritis: insight on subclinical pelvic
inflammatory disease
Harold C. Wiesenfeld, MD, CM
2:00 pm Vaginal polymorphonuclear leukocytes and bacterial vaginosis as markers for
acute endometritis
Mark Yudin, MD
2:15 pm Influences of vaginal and embryo transfer catheter microbiology on early pregnancy
loss following in vitro fertilization
Linda O. Eckert, MD
2:30 pm The risk of acquisition of herpes simplex virus (HSV) during pregnancy:
a prospective couples study
Carolyn Gardella, MD
2:45 pm A double-blind, randomized, placebo-controlled trial of acyclovir in late pregnancy
for reduction of herpes simplex virus (HSV) shedding and cesarean section
D. Heather Watts, MD - BEST ABSTRACT AWARD
3:00 - 4:00 pm Scientific Poster Session
6:00 - 8:00 pm Welcome Reception
Program
INFECTIOUS DISEASES IN OBSTETRICS AND GYNECOLOGY 157
Thursday, August 9, 2001
Scientific Poster Session 3:00 pm
Interaction and Discussion between Poster Presenters and Attendees
AUTHOR AND ABSTRACT
1. Walter Chaim, MD, Soroka Medical Center
Measurement of biogenic amines by ion mobility spectrometry (IMS) as an indication for bacterial
vaginosis (BV)
2. Christine Isaacs, MD, MCV Hospital
Congenital CMV infection presenting as a molar pregnancy
3. Jeanna M. Piper, MD, UTHSC-SA
Direct comparison of Abbott LCX to Genprobe Pace II for gonorrhea and chlamydia
4. Arlene Bardeguez, MD, UMDNJ, New Jersey Medical School
Intracellular IL-4 cytokine production in HIV (+) and HIV (-) women
5. Shivani Singh, MD, Wayne State University
Vaginitis due to Candida krusei: epidemiology, clinical aspects and therapy
6. Michael Leung, MD, University of Texas at Houston
The effect of temperature on bactericidal properties of 10% povidone-iodine solution
7. Jose A. Simoes, MD, PhD, Rush Medical College
Cellulose sulfate inhibits Gardnerellavaginalis and other anaerobes associated with bacterial vaginosis
8. Deborah Cohan, MD, University of California - San Francisco
HPV subtypes, vulvar dysplasia and HIV-infection
9. Lin Tao, DDS, PhD, University of Illinois
Phage infection of lactobacilli: prevalence and host ranges
10. Stella Nowicki, DDS, University of Texas Medical Branch
Serum complement activity during menses as a risk factor for gonococcalpelvic inflammatory disease
11. Stella Nowicki, DDS, University of Texas Medical Branch
Ampicillin-resistant Escherichia coli in gestational pyelonephritis: increased occurrenceand association
with the colonization factor DR adhesin
12. Jeanne Sheffield, MD, University of Texas
Placental histopathology of congenital syphilis correlated to pregnancy outcome
13. James Hill, MD, University of Texas
Efficacy of common antiretroviral regimens during pregnancy
Program
158 INFECTIOUS DISEASES IN OBSTETRICS AND GYNECOLOGY
14. Robert Gutman, MD, Women & Infants Hospital
Evaluation of clinical methods for diagnosing bacterial vaginosis
15. Carol Stamm, MD, University of Colorado
Cervical manipulation and membrane "stripping" associated with stillbirth caused by group B
streptococcus and other perinatal pathogens: cervical membrane disruption syndrome (CMDS)
16. Amy Murtha, MD, Duke University Medical Center
Apoptosis in the chorion laeve of term patients with histologic chorioamnionitis
17. Michael Nicolazzo, BS, University of Pittsburgh
Primary cultures of endocervix and ectocervix demonstrate unique cytokine secretion patterns
18. James McGregor, MD, University of Colorado
Neurologic impairment among offspring of mothers with an intrauterine device (IUD) during
pregnancy: is there an "IUD baby syndrome"?
19. Michel Fortier, MD, University of Laval
Imiquimod 5% cream is safe and effective in female patients with external genital wards: results of
an open-label multi-center trial
Program
INFECTIOUS DISEASES IN OBSTETRICS AND GYNECOLOGY 159
Friday, August 10, 2001
7:00 am - 1:00 pm Registration
8:00 - 9:30 am Scientific Session II Moderator: David Soper, MD
8:00 am Determination of ureaplasma urealyticum biovars in post-partum endometritis (PPE)
Walter Chaim, MD
8:15 am Antimicrobial resistance alert among anaerobes: comparison of ob-gyn and
intra-abdominal (IA) isolates
Kenneth Aldridge, PhD
8:30 am Patterns of sexual activity among high-risk minority women: implications for topical
microbicide development
Jeanna Piper, MD
8:45 am Vaginal microflora of sexually inexperienced women: impact of sexual debut
Sharon Hillier, PhD
9:00 am Demographic and behavioral risk factors for losing vaginal colonization with H2O2+
lactobacillus spp
Marijane Krohn, PhD
9:15 am Interaction between lactobacilli and gardnerella vaginalis in vitro as a key in the
pathogenesis of bacterial vaginosis
Alla Aroutcheva, MD, PhD
9:30 - 9:45 am Break
9:45 - 11:15 am Scientific Session III Moderator: Phil Heine, MD
9:45 am Does second trimester amniotic fluid interleukin-6 and interleukin-10 concentrations
predict preterm delivery?
Joseph Apuzzio, MD
10:00 am Tumor necrosis factor alpha (TNF ) down-regulates oxytocin receptor binding in
cultured human uterine myocytes
Phillip Rauk, MD
10:15 am Tumor necrosis factor-alpha (TNF-alpha) concentration in amniotic fluid and fetal
death in a rabbit model of chronic infection
Mirjam Kunze, MD - YOUNG INVESTIGATOR AWARD
10:30 am Decreased cervical proinflammatory cytokines in early pregnancy are associated with
subsequent intraamniotic infection
Hyagriv Simhan, MD
10:45 am Relationship between maternal and fetal antibodies to vaginal anaerobes and
preterm birth
Kim Boggess, MD
11:00 am Does the clinical setting influence ob/gyn patient vaccine awareness?
Bernard Gonik, MD
11:15 - 11:45 am "Chlamydia screening of young women: update on the current practice and
interventions to increase screening"
Kathleen Irwin, MD
Centers for Disease Control, Division of STD Prevention
11:45 am - 12:00 noon Break
12:00 noon - 1:00 pm IDSOG Business Meeting
6:00 - 10:00 pm Annual Society Banquet & Awards Presentation
Program
160 INFECTIOUS DISEASES IN OBSTETRICS AND GYNECOLOGY
Saturday, August 11, 2001
7:00 am - 12:00 noon Registration
8:00 - 9:30 am Posters/Breakfast
General session
9:30 - 10:00 am "Update on the 2001 STD Treatment Guidelines"
Kimberly Workowski, MD
Centers for Disease Control, Division of STD Prevention
10:00 - 11:30 am Scientific Session IV Moderator: Lindsay Alger, MD
10:00 am Point mutation in Escherichia coli DR fimbriae abolishes type IV
collagen-binding and renal persistence in experimental
chronic pyelonephritis
Bogdan Nowicki, MD, PhD
10:15 am Perioperative antimicrobial prophylaxis can be improved with regard to
urinary tract infection by means of a long acting cephalosporin as ceftriaxon
Udo Hoyme, MD
10:30 am Progression of periodontal disease during pregnancy is associated
with an increased risk for preterm delivery
Amy Murtha, MD
10:45 am Histologic examination of the umbilical cord as an indicator of intrauterine
inflammation and neonatal morbidity among preterm infants
Carolyn Gardella, MD
11:00 am Cerebral palsy, Escherichia coli and group B streptococcus
Helen McDonald, PhD
11:15 am "Real world" compliance with strategies to prevent early onset GBS
Laura Riley, MD
11:30 am Closing remarks
Sebastian Faro, MD, PhD, IDSOG President
Adjournment
12:00 noon - 2:00 pm IDSOG Council Meeting Luncheon
2:00 - 4:00 pm Industry and IDSOG Meeting
Developing Education & Research In Infectious Diseases For Women
Program
INFECTIOUS DISEASES IN OBSTETRICS AND GYNECOLOGY 161

				

